# High-Temperature Tensile and Creep Properties of Highly Strong Heat-Elongated Polypropylene

**DOI:** 10.3390/polym18040469

**Published:** 2026-02-12

**Authors:** Karin Onaka, Hiromu Saito

**Affiliations:** Department of Applied Chemistry, Tokyo University of Agriculture and Technology, Koganei-shi 184-8588, Tokyo, Japan; s237824q@st.go.tuat.ac.jp

**Keywords:** polypropylene, tensile property, creep property, high temperature, deterioration, deformation behavior

## Abstract

We investigated the high-temperature tensile and creep properties of highly strong heat-elongated polypropylene (elongated PP) before and after long annealing for 21 days at a high temperature of 120 °C. Despite the thermal deterioration caused by the long annealing, the elongated PP exhibited high tensile strength. The yield stress values of the elongated and long-annealed (LA)-elongated PP obtained from engineering stress–strain curves were 60 MPa and 102 MPa, respectively, at 120 °C, whereas that of the unelongated PP was 8 MPa. Due to the suppression of crystalline chain motion at high temperature caused by the presence of crystalline fibrils connected to lamellae, as indicated by the high elastic modulus observed using a dynamic mechanical analyzer, the elongated PP also exhibited excellent high-temperature creep properties despite thermal deterioration. Small-angle X-ray scattering and DSC measurements revealed that lamellae were fragmented in the elongated PP, while the fragmentation of lamellae was suppressed in the LA-elongated PP during tensile stretching and creep. These characteristic deformation behaviors might also provide excellent high-temperature properties. The excellent high-temperature properties of the elongated PP are promising for industrial applications that require resistance to high temperatures.

## 1. Introduction

Crystalline polymers, such as polypropylene (PP), are used extensively in various applications in daily life and in the field of engineering, including consumer products, automotive parts, medical devices, and film packaging, due to their light weight, chemical resistance, ease of processing, and low cost [1,2,3,4]. Automotive polymer parts [5,6], medical devices that are sterilized at 121 °C under saturated moisture [7,8], and food packages that are heated by microwaves [9] sometimes require resistance to high temperatures. When exposed to high temperatures for long periods of time, polymer materials undergo thermal degradation via oxidation [10,11]. In PP, the presence of tertiary carbon atoms in the backbone allows oxygen to react, and undergo β-scission with main-chain scission to form the unsaturated structures of vinyl bonds, ketones and aldehydes, or abstract hydrogen without main-chain scission to form hydroxyl groups [12,13,14,15]. Thermal deterioration increases crystallinity by promoting the crystallization of low-molecular-weight chains formed by chain scission [16,17] while decreasing crystallinity due to defects in the crystalline region [18]. Changes in the crystalline structure caused by thermal deterioration affect the performance properties of materials [18,19,20], particularly their tensile properties, and this deterioration leads to embrittlement [10,21] and a decrease in strength [22,23,24]. These unfavorable changes in tensile properties caused by thermal deterioration limit the use of polymer materials at high temperatures.

Creep behavior is also undesirable in polymer materials intended for use at high temperatures. Polymer materials deform under constant loads over time, and the increase in strain becomes larger and faster at elevated temperatures [25,26,27,28]. When materials are subjected to a stress lower than their yield stress, strain changes in several stages during creep: (I) strain increases quickly over time, (II) strain increases gradually and linearly over time, and (III) strain grows rapidly over a short period of time until the materials break [25,29,30]. Break time is usually evaluated as the lifetime of polymer materials. During the rapid increase in strain in stage III, the crystallinity and chain orientation increase due to the rearrangement of the crystalline chains [29,30,31]. It has been reported that creep is accelerated by deterioration [32,33]. Therefore, thermal deterioration is also considered undesirable for high-temperature use due to creep. However, creep is suppressed by deterioration when molecular motion decreases [34,35], crystallinity increases [35] or cross-linking occurs [34,36].

To suppress thermal deterioration, antioxidants are generally used as additives [15,37]. Thermal deterioration is also suppressed by controlling the structure, such as crystallinity [38,39], crystal form [39,40], chain orientation [41,42,43,44] and crosslinking [45]. Decreases in tensile properties are suppressed by suppressing thermal deterioration through chain orientation [44]. Creep is suppressed by increasing crystallinity [37,46] and controlling the crystalline structure [31,47,48,49]. Recently, we found that highly strong elongated PP exhibited a high elastic modulus and high tensile stress at high temperatures; e.g., the yield stress was 60 MPa at 120 °C due to the fragmentation of the lamellae during stretching at small strains and the suppression of crystalline chain motion by crystalline fibrils connected to the lamellae [50]. Due to its high elastic modulus and high yield stress at high temperatures, elongated PP is expected to maintain high tensile and/or creep properties at high temperatures despite thermal deterioration because deterioration is considered to be suppressed by structure control, as described above. Extensive work has been done on the strengthening of crystalline polymers by controlling their chain orientation and crystalline structures [44,51,52,53,54,55,56,57,58,59]. Recently, we found that heat-elongated PP exhibited good high-temperature strength, in which fragmentation of lamellae occurred at small strains below the yield point [50]. However, to the best of our knowledge, no reports have been published for these strengthening crystalline polymers on their high-temperature properties, including the effects of thermal deterioration, though high-temperature properties such as high-temperature creep resistance are important for industrial applications that require resistance to high temperatures.

In this paper, we investigated the tensile and creep properties of highly strong heat-elongated polypropylene (PP) at a high temperature of 120 °C before and after long annealing at the same temperature to verify the possibility of achieving high tensile and creep properties at high temperatures despite thermal deterioration caused by long annealing. To understand these properties, the results of dynamic mechanical analysis (DMA), small-angle x-ray scattering (SAXS) and differential scanning calorimetry (DSC) measurements are presented to discuss changes in structure during tensile and creep behavior.

## 2. Materials and Methods

### 2.1. Materials

Polypropylene (PP) used in this study was isotactic polypropylene supplied by Prime Polymer Co., Ltd., Tokyo, Japan (Prime Polypro J-703GR). The melt flow rate (MFR) of this PP was 9.6 g/10 min.

The PP pellet was compression-molded at 200 °C for 3 min using a hot-press machine (11FD, Imoto Machinery Co., Ltd., Kyoto, Japan) and then quenched in a cold-water bath to obtain a quenched PP film with a thickness of about 230 μm. By annealing the quenched PP film at 120 °C for 1 h, the unelongated PP film was obtained.

The elongated PP film was obtained by heating the quenched PP film to 120 °C; then, it was uniaxially elongated up to an elongation ratio of 600% at a constant rate of 50 mm/min at this temperature using a heat stretching apparatus (Taiatsu Techno Corporation, Tokyo, Japan). The elongated film was immediately cooled to room temperature while under tension to prevent significant chain orientation relaxation.

The unelongated and elongated PP films were annealed at 120 °C for a maximum of 21 days in an oven (DVS403, Yamato Scientific Co., Ltd., Tokyo, Japan) and then cooled to obtain the long-annealed (LA)-unelongated and LA-elongated PP films, respectively. The film specimen was annealed by hanging it under no tension, only by the weight of the specimen itself, during the annealing process.

### 2.2. Methods

#### 2.2.1. Tensile Test

A tensile test was performed by stretching a film specimen in the direction parallel to the direction of elongation at a stretching rate of 10 mm/min at different temperatures using a heat stretching apparatus (Taiatsu Techno Corporation, Tokyo, Japan), as described in our previous paper [50]. Dumbbell-shaped film specimens were prepared for the tensile test using a die-cutter according to ASTM D 1708; i.e., the length and width of the specimens were 35.0 mm and 5.0 mm, respectively. The film specimen was stretched after it was thermally stabilized for 5 min at the stretching temperature. After stretching, the specimen was immediately cooled to room temperature while under tension to prevent significant chain orientation relaxation. Tensile tests were performed at least three times for each data set, and the data set; the largest break strain is presented because these specimens are least susceptible to damage and defects.

#### 2.2.2. FT-IR Measurement

A Fourier transform infrared (FT-IR) spectrum was measured in reflectance mode using an FT-IR spectrometer (FT-IR-4100 type A, JASCO Engineering Co., Ltd., Tokyo, Japan) with an attenuated total reflectance (ATR) method with a resolution of 4.0 cm^−1^ and 32 scans. The FTIR spectra were corrected for ATR penetration depth effects and baseline fluctuations. The corrected spectra were then normalized to the intensity of the CH_3_ associated with the symmetric in-plane bending of CH_3_ in PP at 1450 cm^−1^ [60,61].

#### 2.2.3. DMA Measurement

Dynamic mechanical analysis (DMA) was carried out using a DMA1 (Mettler Toledo, Columbus, OH, USA) in tension mode. The film specimen was 5 mm wide with a gap distance of 5 mm. The specimen was swept from −50 to 140 °C at a heating rate of 2 °C/min, a constant oscillatory frequency of 1 Hz, and an amplitude of 0.2% strain. The storage modulus, *E*′, was obtained as a function of temperature.

#### 2.2.4. SAXS Measurement

A small-angle X-ray scattering (SAXS) experiment was performed using the NANO-Viewer system (Rigaku Co., Ltd., Tokyo, Japan). Cu-Ka radiation with a wavelength of 0.154 nm was generated at 46 kV and 60 mA and was collimated by a confocal max-flux mirror system. An SAXS intensity image was measured at room temperature for an exposure time of 1 h using an imaging plate (IP) (BAS-SR 127, Fujifilm Corp., Tokyo, Japan) as a two-dimensional detector. The X-ray was radiated at the midpoint of the stretched area. The scattering images obtained were transformed into text data by an IP reading device (RAXIA-Di, Rigaku Co., Ltd., Tokyo, Japan). The scattering intensity was corrected for the specimen thickness, the beam transmittance, and background scattering.

#### 2.2.5. DSC Measurement

Dynamic scanning calorimetry (DSC) measurement was performed on the specimen at a heating rate of 10 °C/min in a nitrogen atmosphere using a DSC-Q200 (TA Instruments, New Castle, DE, USA). The weight of the specimens used for the measurement was about 3 mg.

#### 2.2.6. Creep Test

A creep test was performed by measuring the change in length of a film specimen over time in an oven (DVS403, Yamato Scientific Co., Ltd., Tokyo, Japan) at a constant temperature of 120 °C after applying a constant load of 24.8 MPa, using a displacement gauge (DP-500G, Tokyo Measuring Instruments Laboratory Co., Ltd., Tokyo, Japan) with a calibration coefficient of 0.0500 mm/1 × 10^−6^ and a PC-controlled dynamic strain meter (DC-004P, Tokyo Measuring Instruments Laboratory Co., Ltd., Tokyo, Japan). The load of 7.5 N consisted of a weight of 610 g and a tension of 1.5 N on the displacement gauge. The specimen used for the creep test was the same dumbbell-shaped film used for the tensile test, and the specimen was thermally stabilized for 5 min before stretching. After the break, the specimen was retrieved and cooled with water. Creep tests were performed at least three times for each data set.

## 3. Results and Discussion

### 3.1. Change in Tensile Properties Caused by Thermal Deterioration

**Figure 1:**
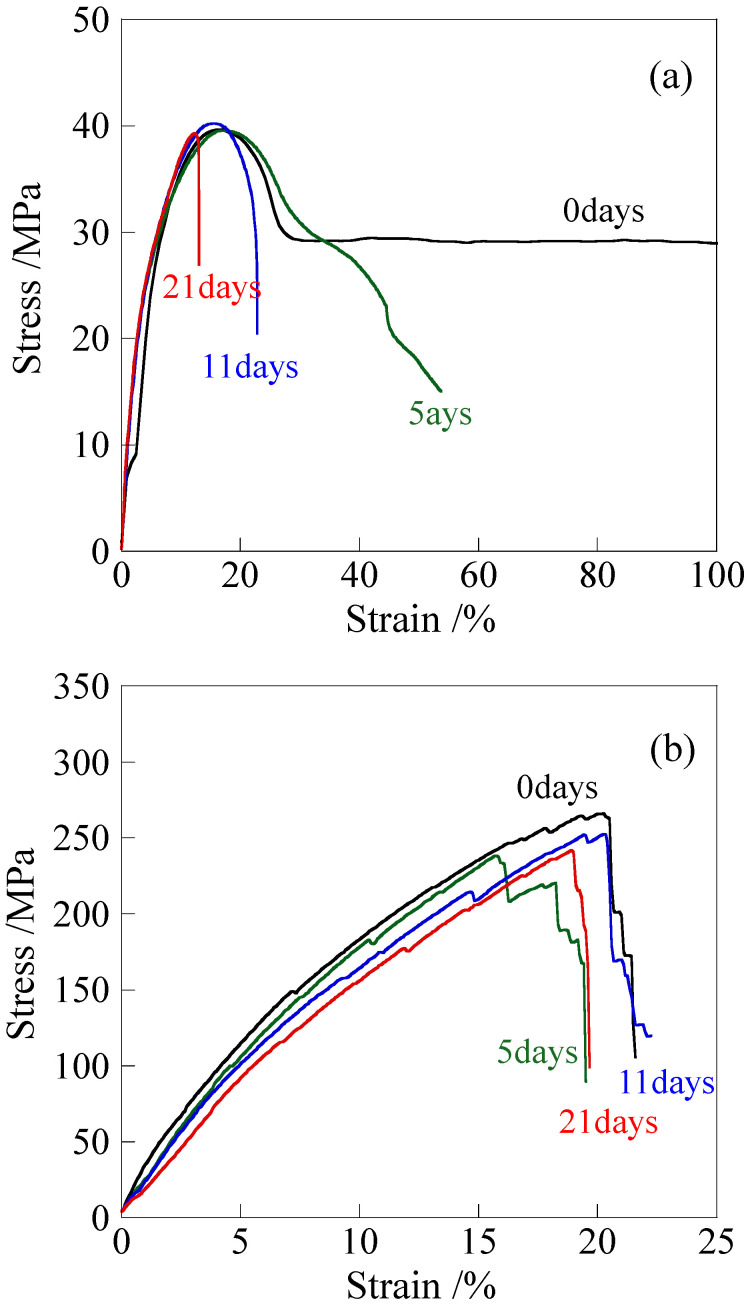
Stress–strain curves measured at room temperature of PP after annealing at 120 °C for various times: (**a**) unelongated PP; (**b**) elongated PP.

Figure 1 shows the engineering stress–strain curves of the unelongated and elongated polypropylene (PP) after annealing at 120 °C for various times, measured at room temperature. The representative stress–strain curves with the same maximal axis values for both diagrams are shown in Figure S1 for comparison. Here, the unelongated PP was obtained by annealing the melt-quenched PP at 120 °C for 1 h, and the elongated PP was obtained by heat elongation of the melt-quenched PP at 120 °C up to an elongation ratio of 600%. The tensile test for the elongated PP was carried out with the stretching direction parallel to the elongated direction. In unelongated PP, the strain at break decreased significantly with an increasing annealing time, and it broke before strain hardening occurred after 5 days; i.e., the break strain values of the unelongated PP after annealing for 0 and 5 days were 753% and 61%, respectively, while the yield stress remained unchanged (Figure 1a). In other words, the tensile properties changed from ductile to brittle. This embrittlement is considered to be caused by a decrease in toughness due to the chain scission caused by thermal deterioration [10,21]. On the other hand, the elongated PP exhibited a high tensile stress of about 266 MPa [50], and no significant changes in the stress and break strain were observed in the elongated PP (Figure 1b). Despite the long annealing for 21 days, the elongated PP exhibited minimal change in break stress, maintaining a high break stress above 240 MPa. Note that a vertical crack occurred in the direction of stretching, followed by macroscopic fibrillation [62] just before the elongated PP broke, as shown in Figure S2. Due to macroscopic fibrillation, stress decreased steeply and discontinuously until the film broke completely. Despite the vertical crack and macroscopic fibrillation, the elongated PP retained high tensile stress. Therefore, the elongated PP retains its high tensile stress even after long annealing. From an engineering standpoint, it should be noted that the materials are considered unusable beyond the strain at which vertical cracks initiate and the stress–strain behavior becomes unstable.

**Figure 2:**
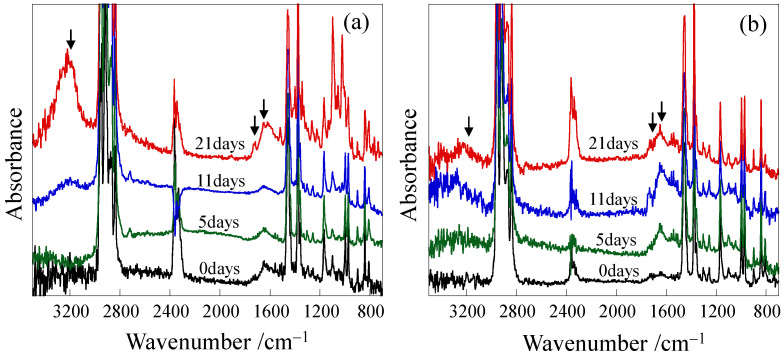
FT-IR spectra of PP after annealing at 120 °C for various times: (**a**) unelongated PP; (**b**) elongated PP. Arrows indicate the peaks for the vinyl (1650 cm^−1^), carbonyl (1720 cm^−1^) and hydroxyl (3200 cm^−1^) groups.

Thermal deterioration caused by annealing at a high temperature of 120 °C was confirmed in both the unelongated and elongated PP using Fourier transform infrared (FTIR) spectra, as shown in Figure 2. Peaks appeared at around 1650 cm^−1^, 1720 cm^−1^ and 3200 cm^−1^ after long annealing in the unelongated PP (Figure 2a). The peaks that appeared at around 1650 cm^−1^, 1720 cm^−1^ and 3200 cm^−1^ are attributed to the formation of vinyl groups, carbonyl groups and hydroxyl groups, respectively, which result from the thermal deterioration of PP due to oxidation [13,63,64,65]. Aldehydes and ketones containing carbonyl groups and an unsaturated structure with vinyl bonds are formed by β-scission with main-chain scission, while hydroxyl groups are formed without main-chain scission [13,14,15]. Therefore, the embrittlement of the unelongated PP caused by high-temperature annealing, as shown in Figure 1a, is confirmed to be caused by thermal deterioration due to oxidation. Significant increases in absorbance with annealing times were also observed at around 1650 cm^−1^, 1720 cm^−1^ and 3200 cm^−1^ in the elongated PP, indicating that thermal deterioration due to oxidation also occurs in the elongated PP, despite no significant change being observed in the tensile properties. For example, the hydroxyl indices of the unelongated and elongated PP after annealing for 21 days—obtained by calculating the ratio of the peak areas at around 3200 cm^−1^ for the hydroxyl group and 1450 cm^−1^ for the C-H bond [60]—were 4.82 and 1.58, respectively. Thus, the high tensile stress remaining in the elongated PP despite long annealing, as shown in Figure 1, cannot be attributed to its resistance to thermal deterioration due to oxidation.

**Figure 3:**
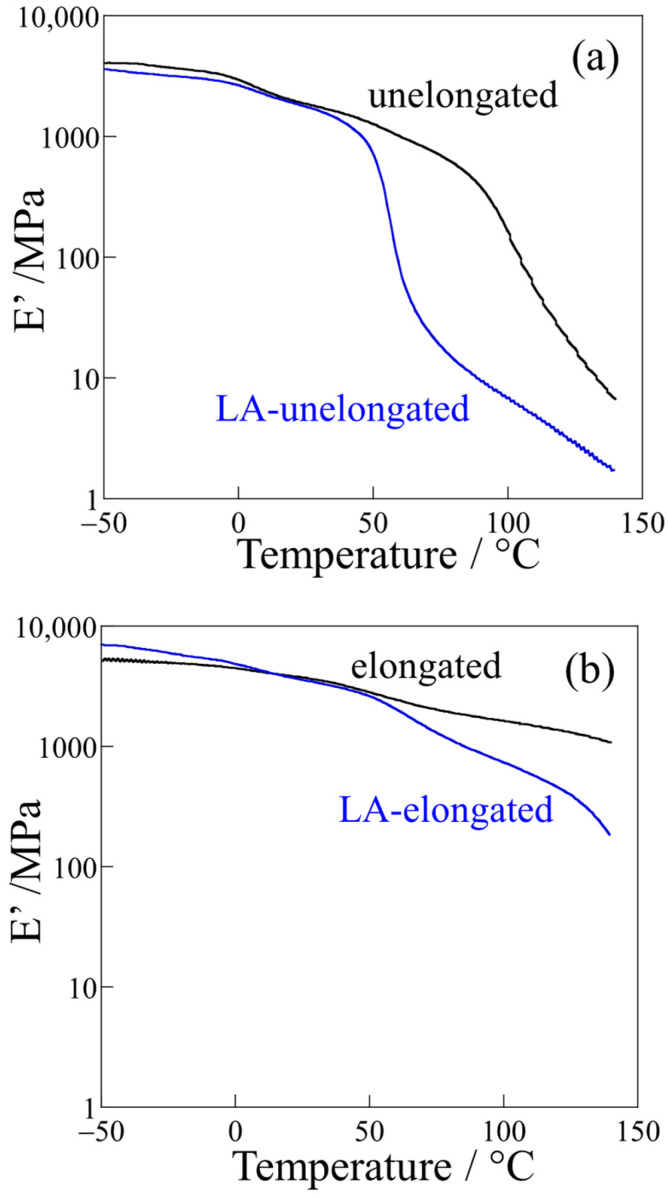
DMA thermograms: (**a**) unelongated PP; (**b**) elongated PP.

Figure 3 shows the elastic modulus (*E*′) with temperature obtained by dynamic mechanical analysis (DMA) at a frequency of 1 Hz for the unelongated and elongated PP before and after long annealing for 21 days at a high temperature of 120 °C. In the unelongated PP, the *E*′ decreased steeply at around 90 °C due to the crystalline chain motion caused by αc-relaxation (Figure 3a). The decrease in *E*′ was greatly accelerated in the LA-unelongated PP, and the steep decrease started to occur at around 50 °C. This acceleration is attributed to the increased chain motion caused by the chain scission due to thermal deterioration. The decrease in *E*′ due to the onset of crystalline chain motion caused by αc-relaxation was suppressed in the elongated PP (Figure 3b). Here, αc-relaxation, which occurs at the inflection points in Figure 3, is characteristic of crystalline chain motion, which is caused by the slippage of crystalline chains [66,67] and the diffusion of conformational defects within the crystals [20,68,69]. This suppression is due to the presence of crystalline fibrils connected to lamellae, as described in our previous paper [50]. An accelerated decrease in *E*′ was also observed in the LA-elongated PP due to increased chain motion caused by the chain scission due to thermal deterioration. However, this decrease was smaller in the LA-elongated PP compared to that of the LA-unelongated PP, and a high *E*′ remained at high temperatures in the LA-elongated PP; e.g., the *E*′ values of the LA-unelongated PP and LA-elongated PP were 4 MPa and 455 MPa, respectively. These results indicate that the increased chain motion caused by thermal deterioration is suppressed in the LA-elongated PP, and the LA-elongated PP retains its high tensile properties at high temperatures. The smaller decrease in the *E*′ of the LA-elongated PP might be due to the presence of crystalline fibrils connected to lamellae, as was indicated in the elongated PP.

### 3.2. High Temperature Tensile Properties

**Figure 4:**
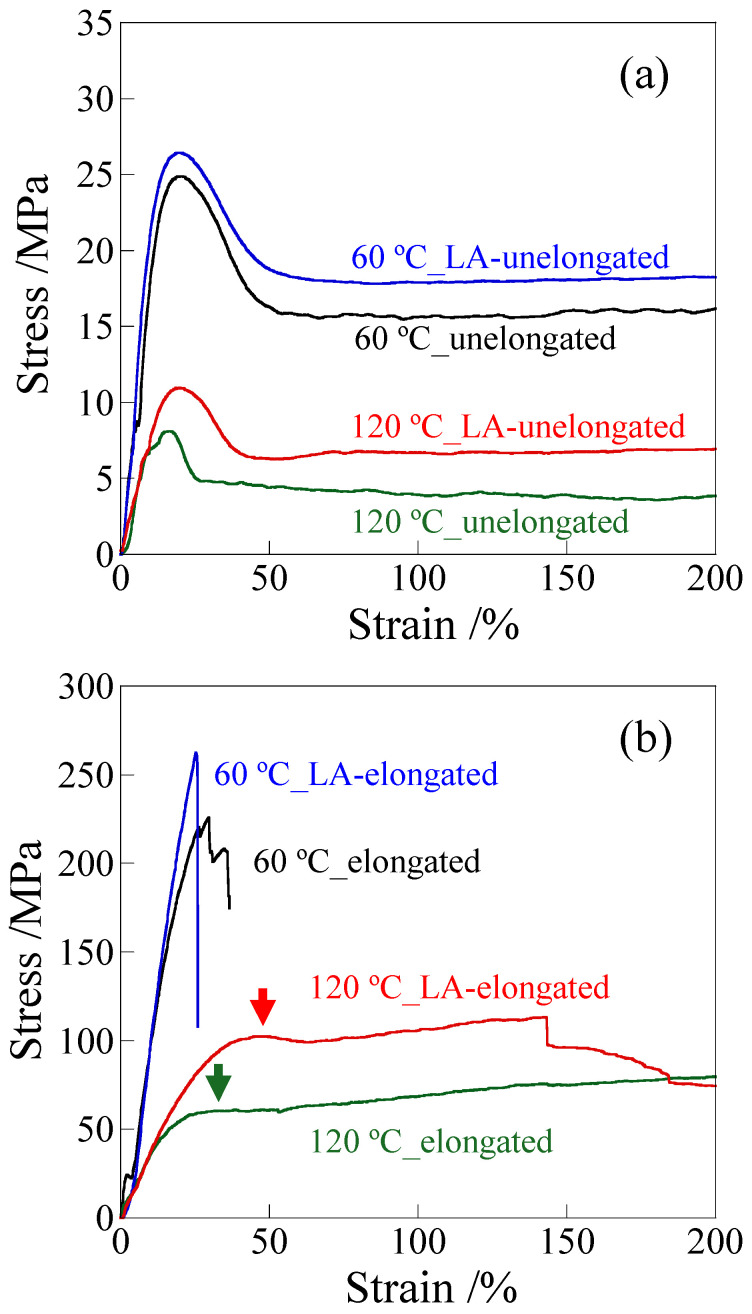
Stress–strain curves measured at different temperatures: (**a**) unelongated PP; (**b**) elongated PP.

Figure 4 shows the engineering stress–strain curves of the unelongated and elongated PP before and after long annealing at 120 °C for 21 days, at different stretching temperatures. Here, the tensile test for the elongated PP was performed by stretching it parallel to the elongated direction. The brittle property of the LA-unelongated PP at room temperature (Figure 1a) changed to a ductile property at high temperatures due to increased crystalline chain motion. The yield stress of the LA-unelongated PP was slightly higher than that of the unelongated PP; i.e., the yield stresses of the LA-unelongated PP and unelongated PP at 120 °C were 11 MPa and 8 MPa (Figure 4a), although the *E*′ of the LA-unelongated PP was much lower than that of the unelongated PP due to thermal deterioration (Figure 3a). The higher yield stress of the LA-unelongated PP might be due to its higher crystallinity, as suggested in recycled PP [70]; i.e., the melting enthalpy, Δ*H*, values of the LA-unelongated PP and unelongated PP obtained from the area of the melting peak were 123.9 J/g and 108.5 J/g, respectively (Figure S3).

**Figure 5:**
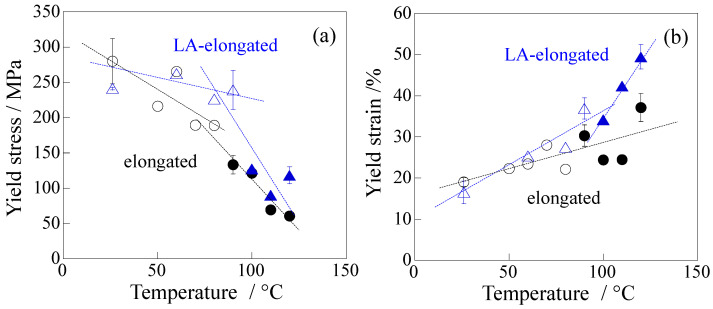
Tensile properties of the elongated PP and LA-elongated PP at various temperatures: (**a**) yield stress; (**b**) yield strain. White-filled plots indicate the specimens in which vertical cracks occurred before reaching the yield point.

In the elongated PP, the stress of the LA-elongated PP was higher than that of the elongated PP before long annealing (Figure 4b). The yield stress of the LA-elongated PP was 1.7 times higher than that of the elongated PP; i.e., the yield stresses of the LA-elongated PP and elongated PP at 120 °C were 102 MPa and 60 MPa, respectively (as indicated by the arrows). Thus, the high tensile stress of elongated PP remained despite the thermal deterioration caused by long annealing, though a steep drop was observed at around a strain of 140% due to macroscopic fibrillation. Similar to the results for the unelongated PP, the yield stress of the LA-elongated PP was higher than that of the elongated PP, although the *E*′ of the LA-elongated PP was lower than that of the elongated PP (Figure 3b). However, the higher yield stress of the LA-elongated PP cannot be explained by the difference in crystallinity because the crystallinity of the LA-elongated PP was lower than that of the elongated PP; i.e., the Δ*H* values of the LA-elongated PP and elongated PP were 130.6 J/g and 150.9 J/g, respectively (Figure S3). In the LA-elongated PP, the yield stress and yield strain increase to higher values simultaneously, so that the high yield stress is caused by an increase in yield strain. Similar results were also observed at other temperatures; i.e., both the yield stress and yield strain of the LA-elongated PP were higher than those of the elongated PP (Figure 5). Therefore, the higher yield stress of the LA-elongated PP is attributed to its characteristic structure change during tensile stretching, which differs from that of the elongated PP, as demonstrated below.

**Figure 6:**
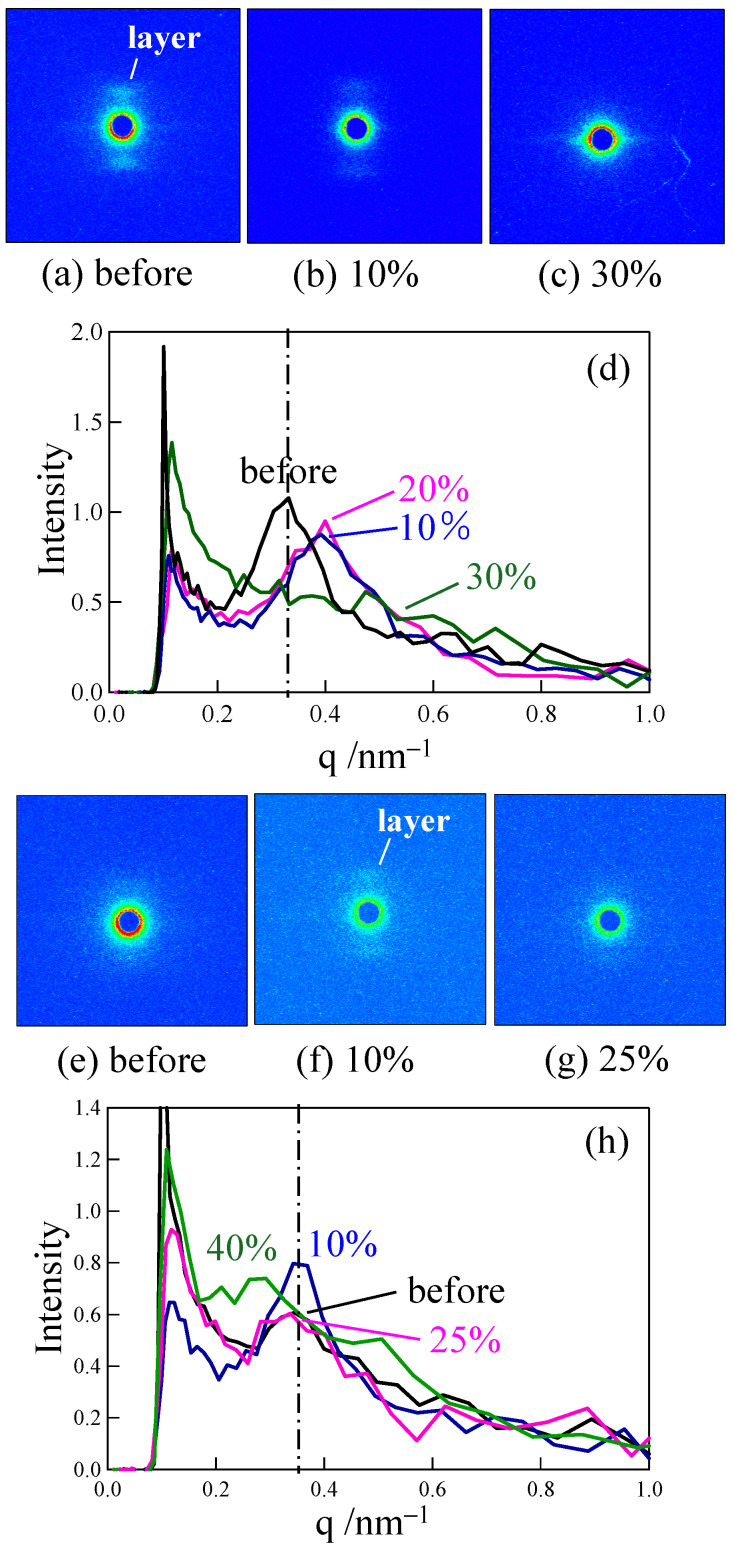
SAXS images and intensity profiles of (**a**–**d**) elongated PP and (**e**–**h**) LA-elongated PP, obtained after stretching up to various strains at 120 °C. The intensity profile was obtained by integrating the scattering intensity within the azimuthal angle region of 65° to 115° from the 2D intensity distribution of the SAXS image (Elongated direction was 90°).

Figure 6 shows the changes in small-angle X-ray scattering (SAXS) images and the intensity profiles when stretched at 120 °C up to a small strain around the yield point for the elongated PP and LA-elongated PP. A clear layer pattern attributed to the macroscopically arranged lamellar stacks changed to a diffuse layer pattern when stretched to a small strain in the elongated PP (Figure 6a–c). A peak in the intensity profile broadened, and the peak position shifted to a higher scattering vector, *q* (Figure 6d), suggesting that the lamellae are fragmented, and the arrangement of lamellae is disordered during stretching at a small strain below the yield point, as described in our previous paper [50]. On the other hand, a diffuse layer pattern changed to a clear layer pattern during stretching (Figure 6e,f), and then, the layer pattern changed back to a diffuse layer pattern in the LA-elongated PP (Figure 6f,g). Along with the change in the SAXS pattern, a broad peak in the intensity profile changed to a sharp peak and then broadened again without a significant change in peak position as the strain increased to 25% (Figure 6h). The absence of a significant change in the peak position indicates that the periodic distance between adjacent lamellae, i.e., the long period, did not change significantly due to the suppression of lamellar fragmentation. A broad peak remained, and the peak position shifted to a lower *q* at a strain of 40%, which was close to the yield strain, suggesting that the periodic distance between the adjacent lamellae increases with strain, while the lamellar stacks remain due to the suppression of lamellar fragmentation. These results suggest that the irregularly arranged lamellae in the LA-elongated PP changed to regularly arranged ones by associating with the arrangement of the relaxed tie chains and crystalline fibrils, and then, the lamellae became irregularly arranged due to their partial fragmentation, while the stacked lamellae remained due to the suppression of lamellar fragmentation.

**Figure 7:**
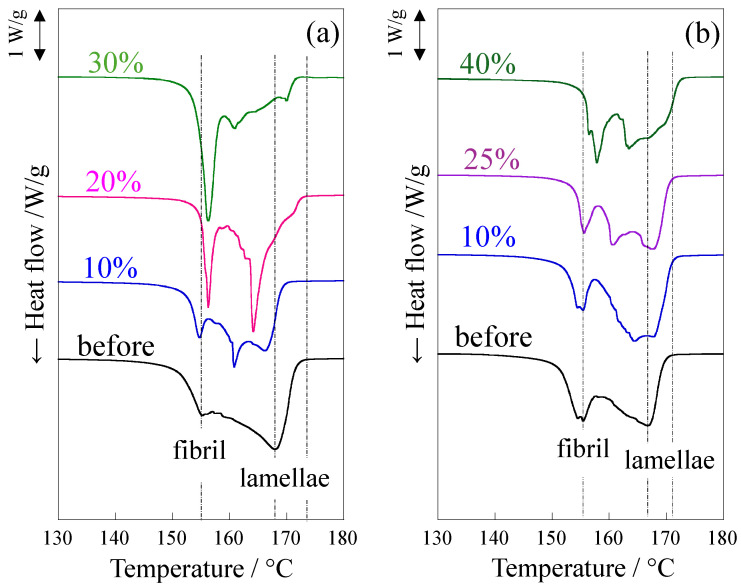
DSC thermograms for melting peak of (**a**) elongated PP and (**b**) LA-elongated PP, obtained after stretching up to various strains at 120 °C. The DSC curves are vertically shifted.

Figure 7 shows the changes in the melting peaks of DSC thermograms when the elongated and LA-elongated PP were stretched at 120 °C up to a small strain around the yield point. Two peaks were observed before stretching due to the co-existence of lamellae and crystalline fibrils. The higher and lower melting peaks around 155 °C and 168 °C are assigned to thicker lamellae and thinner crystalline fibrils, respectively [50]. Note that the DSC curves of the LA-elongated PP are shown at a higher strain than those of the elongated PP because the yield strain of the LA-elongated PP was higher than that of the elongated PP, as shown in Figure 4b. The higher temperature peak shifted significantly to lower temperatures and then disappeared because of stretching, suggesting that lamellae were fragmented during stretching below the yield point in the elongated PP (Figure 7a). On the other hand, the change in the higher temperature peak was slight in the LA-elongated PP (Figure 7b), suggesting that the fragmentation of lamellae is suppressed, as indicated by the SAXS results shown in Figure 6. At a strain of 25%, two melting peaks changed to three melting peaks owing to the appearance of a melting peak at a temperature between the pre-existing higher and lower melting peaks. This suggests that thick lamellae are partially fragmented while a large number of lamellae remain due to the suppression of lamellar fragmentation, and the change to irregularly arranged lamellae is due to partial fragmentation of lamellae. At a strain of 40%, close to the yield strain, the higher melting peak shifted to a lower temperature, suggesting that the thick lamellae are further fragmented. Therefore, the irregularly arranged stacked lamellae in the LA-elongated PP change to regularly arranged ones by suppressing lamellar fragmentation and then change to irregularly arranged ones by partially fragmenting the lamellae. The suppression of lamellar fragmentation and the subsequent partial fragmentation of lamellae in the LA-elongated PP might result in an increase in yield strain, consequently increasing yield stress at high temperatures. The strengthening caused by the suppression of lamellar fragmentation in the LA-elongated PP is consistent with the tensile behavior observed in high-density polyethylene [57] and thermoplastic polyurethane [58], but it differs from the strengthening mechanism of the elongated PP in which the strengthening occurs owing to the fragmentation of lamellae and the presence of fine crystals [50,53,59,71]. The lower temperature peak attributed to crystalline fibrils did not disappear during the stretching of either the elongated or LA-elongated PP up to the yield point, indicating that the crystalline fibrils remained intact during stretching. The presence of crystalline fibrils connected to lamellae in the elongated and LA-elongated PP suppresses the decrease in stress at high temperatures, resulting in high tensile strength at high temperatures, as shown in Figure 3, Figure 4 and Figure 5.

### 3.3. High-Temperature Creep Properties

**Figure 8:**
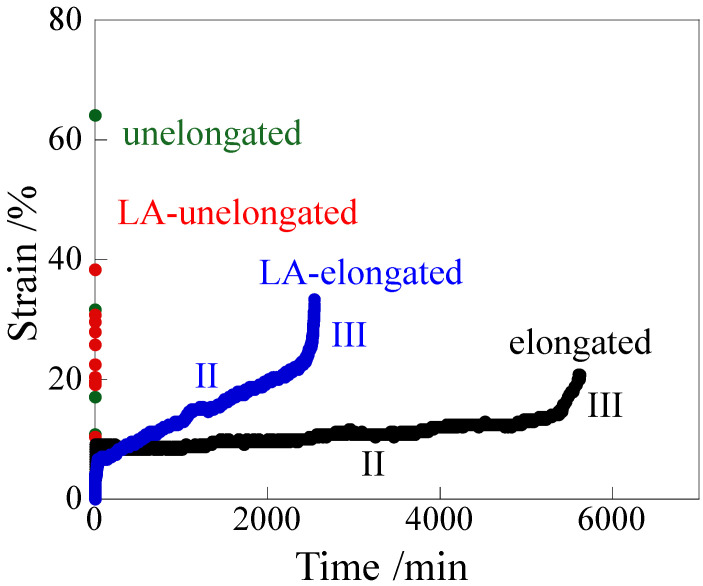
Creep curves for the unelongated and elongated PP before and after annealing under a load of 24.8 MPa at 120 °C. II and III indicate the stages of creep.

Figure 8 shows the change in strain over time during creep under a load of 24.8 MPa at a high temperature of 120 °C for both unelongated and elongated polypropylene (PP) with a thickness of 60 μm before and after long annealing at 120 °C for 21 days. The unelongated PP quickly elongated to a large strain. The LA-unelongated PP also quickly elongated, but it broke at a small strain within 1 s (Figure S4). Note that Chang et al. reported that unelongated PP consisting of spherulites quickly elongated at 120 °C at a load above 10 MPa, while a three-stage increase was observed at a load below 9 MPa over a period of 10 min [30]. These results indicate that unelongated and LA-unelongated PP exhibit poor creep resistance at a high temperature of 120 °C under a load of 24.8 MPa. On the other hand, a three-stage increase in strain was observed over a long period of several thousand minutes in the heat-elongated and LA-elongated PP. After an initial steep increase in strain (stage I), strain increased gradually and linearly over time (stage II) and then increased steeply and finally broke (stage III). The strain rate in stage II, derived from the slope, was much slower in the elongated PP than in the LA-elongated PP. Both specimens broke before necking occurred. The increase in strain in region II of the LA-elongated PP was larger than that of the elongated PP, such that the LA-elongated PP broke faster than the elongated PP; i.e., the elongated and LA-elongated PP broke at about 5600 min and 2500 min, respectively. Although the LA-elongated PP broke faster than the elongated PP, the LA-elongated PP broke much slower than the LA-unelongated PP; i.e., the beak times of the LA-unelongated and LA-elongated PP were about 1 s and 2500 min, respectively. Thus, the elongated PP exhibited excellent creep properties at a high temperature despite thermal deterioration caused by long annealing. Creep is considered to be suppressed by a decrease in molecular motion [34,35]. Therefore, the excellent high-temperature creep properties of the elongated PP can primarily be attributed to the large *E*′ at high temperatures (Figure 3b) due to the suppression of crystalline chain motion caused by the presence of crystalline fibrils that are connected to the lamellae. The slower strain rate in stage II of the elongated PP compared to the LA-elongated PP might be due to the larger *E*′ in the elongated PP.

**Figure 9:**
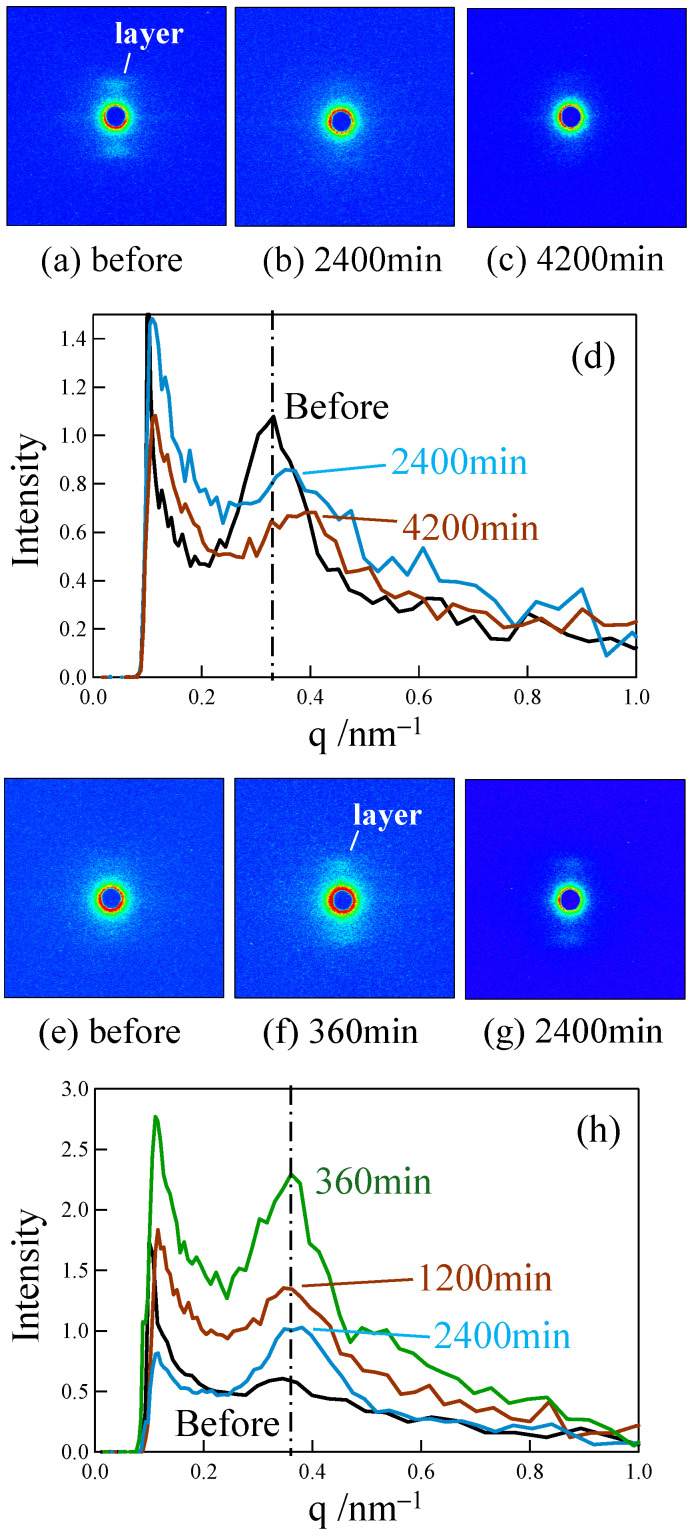
SAXS images and intensity profiles of (**a**–**d**) elongated PP and (**e**–**h**) LA-elongated PP, obtained after creep at various times under a load of 24.8 MPa at 120 °C. The intensity profile was obtained by integrating the scattering intensity within the azimuthal angle region of 65° to 115° from the 2D intensity distribution of the SAXS image (elongated direction was 90°).

To understand the excellent creep properties of the elongated and LA-elongated PP, small-angle X-ray scattering (SAXS) images and intensity profiles were obtained for the elongated PP and LA-elongated PP after creep at 120 °C for various times. The results are shown in Figure 9. The layer pattern attributed to the macroscopically arranged lamellar stacks changed to a diffuse layer pattern with time in the elongated PP in stage II (Figure 9a–c). A peak in the intensity profile broadened, and the peak position shifted to a higher scattering vector, *q* (Figure 9d). The results indicate that the lamellae are fragmented, and the lamellar arrangement becomes disordered with a slight increase in strain in stage II. This structure change is similar to that observed during stretching, as shown in Figure 6a–c. On the other hand, a diffuse layer pattern changed to a clear layer pattern in the LA-elongated PP (Figure 9e,f), and the clear layer pattern remained during creep in stage II (Figure 9e–g). A broad peak became sharper, and its intensity increased and then decreased over time without a significant change in peak position before the break (Figure 9h). The results indicate that fragmentation of the lamellae is suppressed, and the lamellar arrangement becomes ordered and then disordered with a gradual increase in strain in stage II. Notably, this result shows that a structural change occurred in stage II in the elongated PP, while the structural change is minor in ordinary unelongated PP [29,30]. This structure change is also similar to that observed during stretching, as shown in Figure 6f–h.

**Figure 10:**
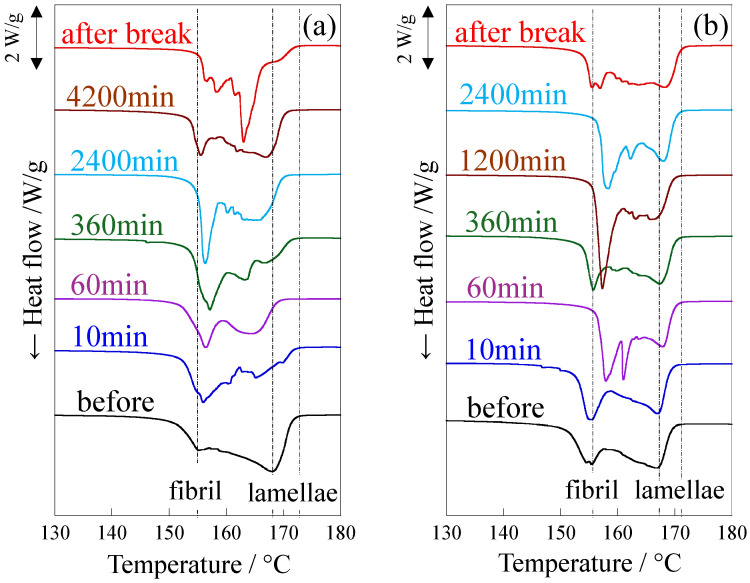
DSC curves for melting peak after creep at various times under a load of 24.8 MPa at 120 °C: (**a**) elongated PP; (**b**) LA-elongated PP. The DSC curves are vertically shifted.

Figure 10 shows the change in the melting peaks in DSC thermograms of the elongated and LA-elongated PP obtained after creep at 120 °C for various times in stage II. The higher temperature peak shifted significantly to lower temperatures due to the fragmentation of lamellae caused by creep in the elongated PP (Figure 10a), while the change in the higher temperature peak was slight, and the higher melting peak did not disappear in the LA-elongated PP (Figure 10b). These results confirm that the decrease in lamellar size was due to lamellar fragmentation in the elongated PP, while the lamellar fragmentation was suppressed in the LA-elongated PP, as suggested by the SAXS results shown in Figure 9. Therefore, lamellae are fragmented, and the lamellar arrangement becomes disordered with a slight increase in strain in stage II in the elongated PP, while fragmentation of lamellae is suppressed, and the lamellar arrangement becomes ordered and then disordered with a gradual increase in strain in stage II in the LA-elongated PP. The lower temperature peak attributed to the crystalline fibrils did not disappear before the break in either the elongated or the LA-elongated PP, indicating that the crystalline fibrils remained intact during creep. The presence of crystalline fibrils connected to lamellae suppresses the crystalline chain motion at high temperatures, resulting in high-temperature creep resistance in elongated and LA-elongated PP, as shown in Figure 8.

### 3.4. Deformation Mechanism During Tensile Stretching and Creep

**Figure 11:**
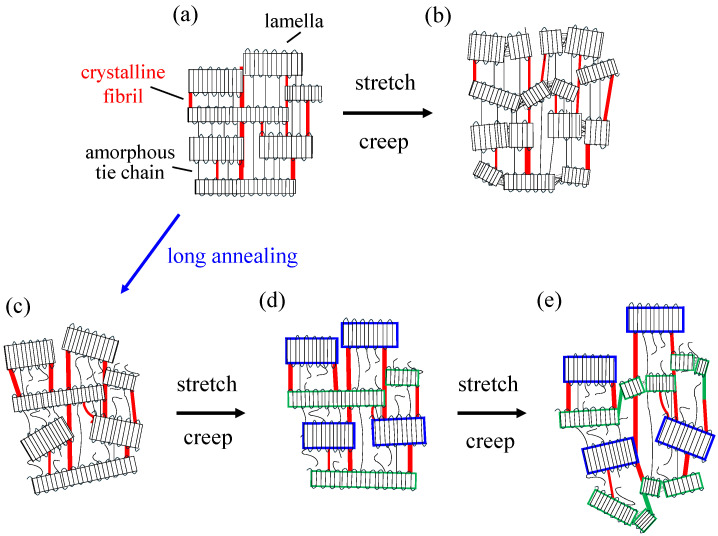
Schematic illustration of the structure change during stretching and creep: (**a**,**b**) elongated PP; (**c**–**e**) LA-elongated PP.

Figure 11 shows a schematic illustration of the deformation mechanism of elongated PP during tensile stretching and creep. The above SAXS and DSC results suggest that the deformation mechanisms of elongated PP during tensile stretching and creep are similar. The elongated PP exhibits macroscopically arranged stacked lamellae consisting of crystalline lamellae and amorphous layers, in which the stacked lamellae are arranged in the elongated direction, and lamellae are connected by amorphous tie chains and crystalline fibrils (Figure 11a). The lamellae are fragmented, and the lamellar arrangement becomes disordered during stretching and creep at a high temperature of 120 °C (Figure 11b). By long annealing for 21 days at a high temperature of 120 °C, the lamellar arrangement in the lamellar stacks becomes disordered due to chain relaxation and chain scission caused by thermal deterioration (Figure 11c). With tensile stretching and creep at a high temperature of 120 °C, the irregularly arranged lamellae can become regularly arranged by elongating the relaxed tie chains and crystalline fibrils connected to the lamellae (Figure 11d). Then, the lamellar stacks become irregularly arranged due to the partial fragmentation of lamellae (indicated by the green color) (Figure 11e). Due to the suppression of the fragmentation of lamellae, associated with the partial fragmentation of lamellae, crystalline fibrils are elongated by the slippage of taut chains within the fibrils. The deformation of LA-elongated PP due to both the partial fragmentation of lamellae and the elongation of crystalline fibrils may result in a higher yield strain and yield stress, causing the yield stress of the LA-elongated PP to be higher than that of the elongated PP, which is deformed by the fragmentation of lamellae (Figure 11b), as shown in Figure 4b. The lamellar stacks remain despite the disordering arrangement of the lamellae due to the suppression of lamellar fragmentation. The suppression of lamellar fragmentation might be attributed to a decrease in transmitted stress to the lamellae due to the scission of tie chains caused by thermal deterioration. The elongated PP and LA-elongated PP exhibit excellent high-temperature tensile and creep properties due to different deformation mechanisms during stretching and creep, as shown in Figure 11a,b and Figure 11c–e, respectively, and due to the presence of crystalline fibrils connected to lamellae, which suppress the crystalline chain motion at high temperatures and do not disappear during stretching and creep.

## 4. Conclusions

We found that highly strong heat-elongated PP (elongated PP) exhibited excellent high-temperature tensile and creep properties despite thermal deterioration caused by long annealing at a high temperature of 120 °C for 21 days. The yield stresses of the elongated and long-annealed (LA)-elongated PP at 120 °C were 60 MPa and 102 MPa, respectively, whereas that of the unelongated PP was 8 MPa. The elongated PP also exhibited excellent high-temperature creep properties despite thermal deterioration; e.g., the elongated PP broke at 120 °C after about 5600 min, while the LA-elongated PP broke after about 2500 min, whereas the LA-unelongated PP broke at 1 s. The excellent high-temperature creep properties of the elongated PP might primarily be attributed to the high elastic modulus at high temperatures observed using DMA due to the suppression of crystalline chain motion caused by the presence of crystalline fibrils connected to the lamellae. SAXS and DSC measurements revealed that crystalline fibrils connected to lamellae remained during stretching and creep, and fragmentation of lamellae and disordered lamellar arrangement occurred in the elongated PP, while fragmentation of lamellae was suppressed, and the lamellar arrangement became ordered and then disordered in the LA-elongated PP. These characteristic deformation behaviors might also provide excellent high-temperature tensile and creep properties. To the best of our knowledge, this is the first report presenting the excellent high-temperature tensile and creep properties of filler-free crystalline polymers. These excellent high-temperature tensile and creep properties, despite thermal deterioration, are promising for industrial applications, such as automobile parts, medical devices, and food packaging, which require resistance to high temperatures and a long lifetime; e.g., the creep lifetime of elongated PP at 120 °C was several thousand minutes under a load of 24.8 MPa, while that of ordinary PP was several tens of minutes under a load of 8 MPa [30]. Simultaneous measurement of stress and birefringence during stress relaxation [72,73] is promising for clarifying the contributions of stress from different components, such as the crystalline and amorphous components of elongated PP at different temperatures.

## Data Availability

The original contributions presented in this study are included in the article and Supplementary Materials. Further inquiries can be directed to the corresponding author.

## Supplementary Materials

The following supporting information can be downloaded at: https://www.mdpi.com/article/10.3390/polym18040469/s1, Figure S1: Stress-strain curves measured at room temperature of unelongated and elongated PP after annealing at 120 °C for various times.; Figure S2: Stress-strain curves measured at room temperature and pictures of specimens at different stages during stretching of elongated PP.; Figure S3: DSC thermograms for melting peak of unelongated and elongated PP before and after annealing at 120 °C.; Figure S4: Creep curves for the unelongated and LA-unelongated PP.
