# Supplementary material for: High-Temperature Tensile and Creep Properties of Highly Strong Heat-Elongated Polypropylene
Source: Polymers (Basel). 2026 Feb 12;18(4):469. doi: 10.3390/polym18040469 (PMC12944040; doi:10.3390/polym18040469)
Supplement: Supplementary file 1 [file polymers-18-00469-s001.zip › polymers-4114604-supplementary.pdf]

Supplementary Materials

High temperature tensile and creep properties of highly strong heat-elongated polypropylene

Karin Onaka, Hiromu Saito

Department of Applied Chemistry, Tokyo University of Agriculture and Technology, Koganei-shi, Tokyo 184-8588, Japan

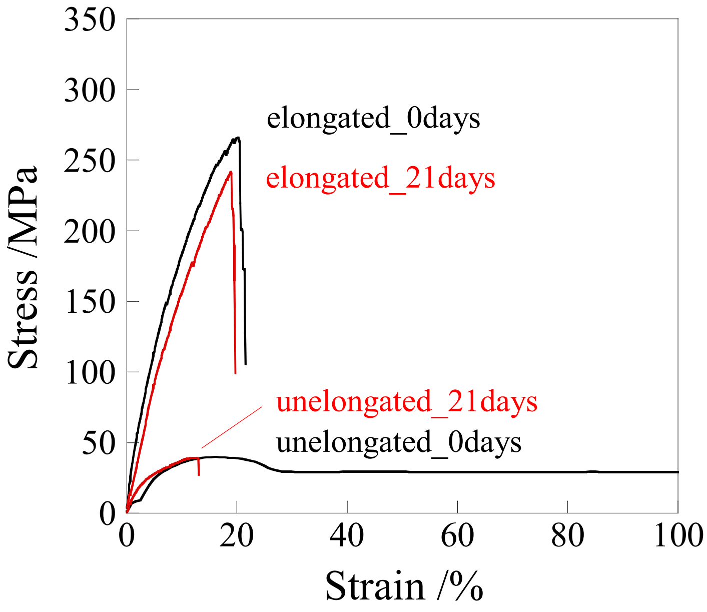

Figure S1. Stress-strain curves measured at room temperature of unelongated and elongated PP after annealing at 120 ºC for various times.

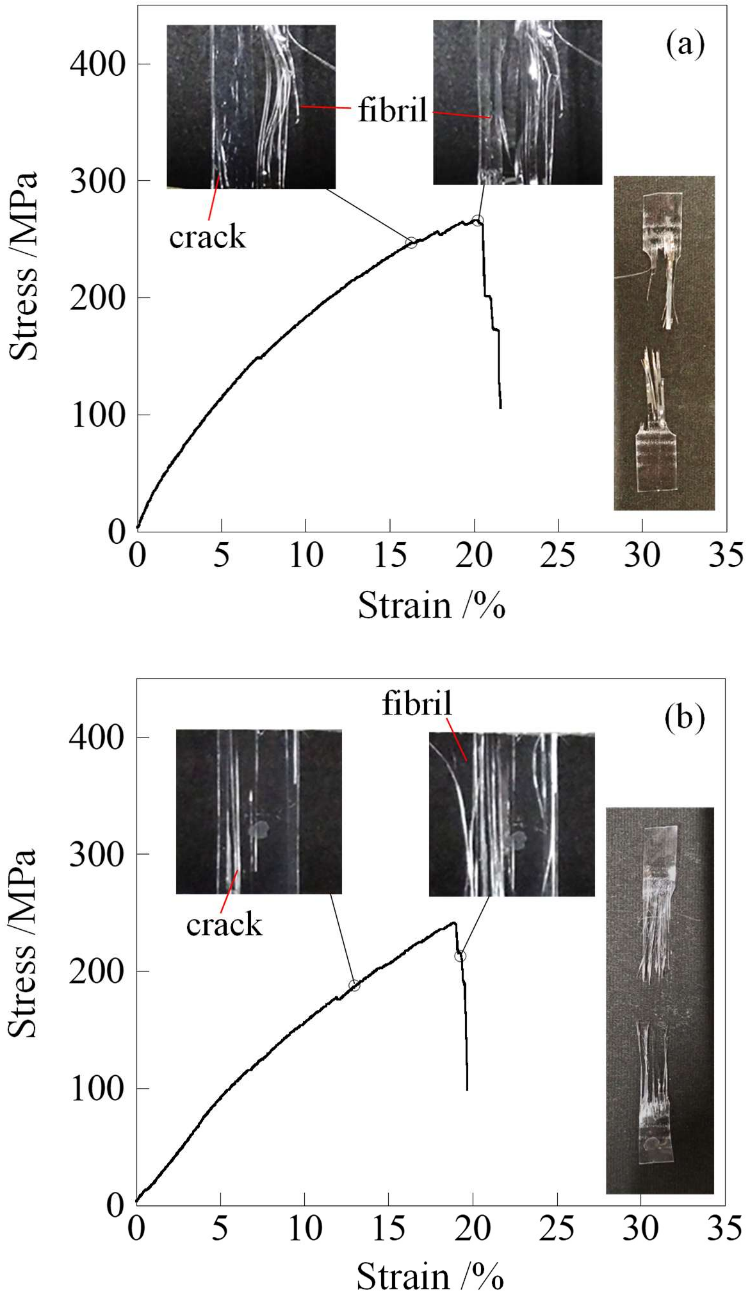

Figure S2. Stress-strain curves measured at room temperature and pictures of specimens at different stages during stretching of elongated PP (a) before and (b) after annealing at 120 ºC for 21days.

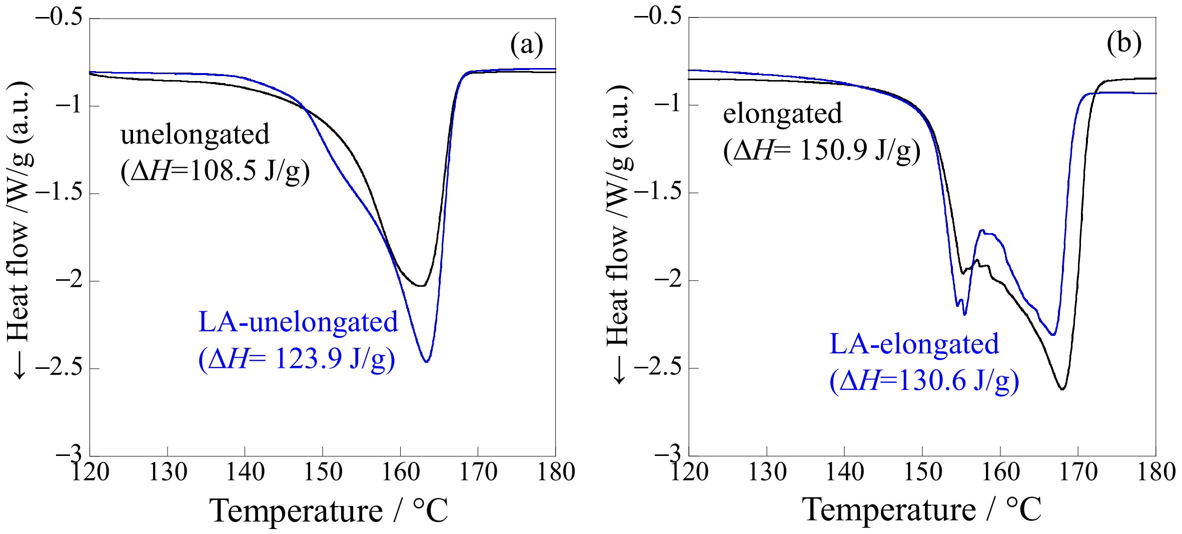

Figure S3. DSC thermograms for melting peak of (a) unelongated and (b) elongated PP before and after annealing at 120 ºC. Heat of fusion Δ*H* was obtained from the area of the melting peak.

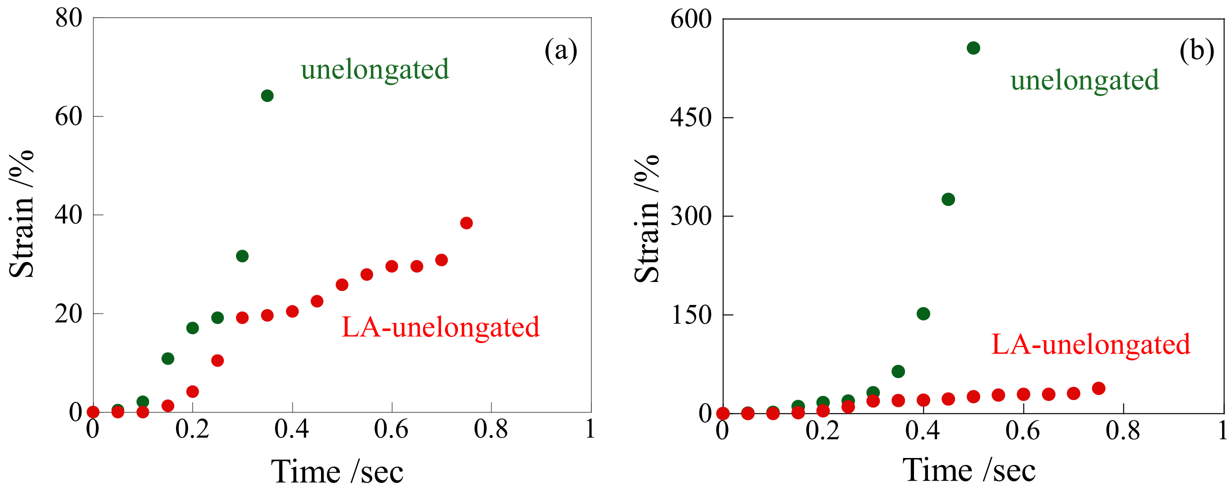

Figure S4. Creep curves for the unelongated and LA-unelongated PP:
(a) small strain, (b) large strain.
